# Adolescent girls’ attitudes toward female genital mutilation: a study in seven African countries

**DOI:** 10.12688/f1000research.14142.1

**Published:** 2018-03-20

**Authors:** Koustuv Dalal, Zhanna Kalmatayeva, Sourav Mandal, Gainel Ussatayeva, Ming Shinn Lee, Animesh Biswas

**Affiliations:** 1 Higher School of Public Health, Al-Farabi Kazakh National University, Almaty, Kazakhstan; 2 Dr. Kanailal Bhattacharya College, Ramrajatala, Howrah, West Bengal, India; 3 National Dong Hwa University, Hualien, Taiwan; 4 Centre for Injury Prevention and Research, Dhaka, Bangladesh

**Keywords:** female genital cutting, attitudes, Egypt, Guinea, Kenya, Mali, Niger, Senegal, Sierra Leone.

## Abstract

**Background: **The study’s aim is to examine adolescent girls’ attitudes toward the continuation or discontinuation of female genital mutilation (FGM) in association with their demographics in seven different countries in Africa.

**Methods: **Data from the women’s survey of the Demographic and Health Surveys (DHS) conducted by the respective ministries (of Health and Family Welfare) in Egypt, Guinea, Kenya, Mali, Niger, Senegal and Sierra Leone were used. Adolescent girls (15–19 years) were included in the current analysis: Egypt (N=636), Guinea (N=1994), Kenya (N= 1767), Mali (N=2791), Niger (N=1835), Senegal (N=3604), Sierra Leone (N=1237).

**Results: **Prevalence of supporting the continuation of FGM among adolescent girls was in Egypt 58%, Guinea 63%, Kenya 16%, Mali 72%, Niger 3%, Senegal 23%, and Sierra Leone 52%. Being Muslim and having low economic status were significantly associated with supporting the continuation of FGM in five of the participating countries. Girls having no education or only primary education in Guinea, Kenya, Mali and Sierra Leone exhibited a higher likelihood of supporting FGM than girls with secondary or higher education. In Egypt, Niger and Senegal there was no association between education and supporting FGM. The girls who stated that they had no exposure to media showed the higher likelihood of supporting FGM in Guinea, Kenya, and Senegal than those with exposure to media.

**Conclusions: **The current study argues that increasing media coverage and education, and reducing poverty are of importance for shifting adolescent girls’ attitudes in favor of discontinuation of FGM.

## Introduction

Female genital mutilation (FGM) is a major public health problem in some parts of the world, especially in Africa and the Middle East ^1– 3^. It is recognized as a violation of the human rights of girls and women ^3^, and is a violation of the Convention on the Rights of the Child, which has been ratified by all of the countries where FGM is common except Somalia ^4^, as the majority of FGM procedures being carried out on young girls ^5^. FGM has no known health benefits ^5^ and it can cause many health problems immediately and later in life, for example pain, bleeding, infections, and increased risks during childbirth both for the mother and the baby ^2, 6^. The number of victims is estimated to between 100 and 140 million ^1, 3, 7^. Around 3 million girls a year face the risk of FGM ^3, 7^. In the seven countries included in this multi-country study the estimated prevalence of FGM among girls and women between 15 and 49 years is high (over 85%) in Egypt, Guinea, Mali, and Sierra Leone. In Kenya and Senegal it is almost 30% and in Niger 2% ^3^. However, the prevalence could vary much within countries by ethnic group.

The first step toward changing the practice of FGM is to change attitudes toward it, even though this can be difficult and psychologically painful ^8^. Knowing what demographic factors appear to influence attitudes towards FGM can help in deciding on the actions to take to promote changes. It is a question for both governments and the people. Among the governments of the included countries, the prevailing attitude is that FGM should be prevented, and many have signed agreements or passed laws banning it ^7, 9^. Even if such laws exist, they may be badly disseminated in some countries due to a lack of central administration ^7, 9^. Many governments also turn a blind eye to the practice ^1^ or are accused of being slow to act ^9^. For example, half of Gambian Health Care professionals working in rural areas support the continuation of FGM ^10^.

More women than men support the practice ^11, 12^. Social pressure and tradition are the most compelling factors for the continuation of FGM ^12, 13^. There are divergent results on the support for FGM among younger girls. WHO ^14^ emphasizes that support for discontinuation is high among younger women, while Masho and Matthews ^12^ and Sipsma and colleagues ^15^ conclude that younger girls are more supportive of FGM. One reason for this could be their having less experience of circumcision, either their own or that of their daughters ^15^. There are also contradictory results on religious beliefs. Islam was not a significant predictor of a favorable attitude towards FGM in Guinea, although a majority believed that FGM was accepted by their religion ^11^. In western Africa it was a predictor except in Niger and Nigeria ^15^. In Ethiopia being Muslim was a predictor ^12^. One explanation that has been offered is that in Ethiopia religious beliefs are based on transmitted interpretations, not on the original religious texts ^13^. Another divergent predictor of attitudes towards FGM is household wealth. Higher levels of household wealth increased women’s support for discontinuation in Guinea ^11^. In some countries wealth was associated with supporting FGM and in some the opposite ^15^. Media also plays a major role in clarifying doubts and misconceptions about FGM ^13^. Decision-makers, leaders in the community, and religious leaders are important channels for modifying cultural beliefs about FGM ^16^. Women with lack of exposure to mass media supported the continuation of FGM to a higher extent than others ^12^. Higher education however, is the main factor associated with supporting discontinuation of FGM in most of the studies ^11, 12, 15– 17^. Empowerment is also a key factor in the elimination of FGM ^18^, although Afifi ^19^ concludes that only high empowerment combined with high education played a significant role.

A multi-country comparison is a unique opportunity to analyze what demographic factors that are associated with attitudes toward the continuation of FGM. It is especially interesting to focus on young women (girls 15–19 years), a group that will play an important role in future decisions on FGM among young girls, that is, their daughters.

The aim of this study is to examine adolescent girls’ attitudes toward the continuation of female genital mutilation in association with their demographics in seven different countries in Africa.

## Methods

This study was part of the women’s survey of the Demographic and Health Surveys (DHS) conducted by the Ministries of Health and Family Welfare in Egypt, Guinea, Kenya, Mali, Niger, Senegal and Sierra Leone, respectively. Household interviews were performed using the same structured questionnaires (the Women’s Questionnaire) in Egypt DHS 2008 ^20^, Guinea DHS 2012 ^21^, Kenya DHS 2008 ^22^, Mali DHS 2006 ^23^, Niger DHS 2012 ^24^, Senegal DHS 2010–11 ^25^, Sierra Leone DHS 2008 ^26^.

Initially, we identified the countries with high prevalence of FGM in WHO reports ^2, 3^. We then searched in DHS databases for FGM prevalence in the selected countries. Due to language barriers, we have only selected countries with English databases for FGM. Seven countries were ultimately selected for the current study.

The sample for the women’s surveys included women of reproductive age (between 15 and 49 years) from both rural and urban areas. The sampling within each country used the sampling procedure probability proportional to population size (PPS) based on the sizes of the state’s/region’s urban and rural populations, which led to nationally representative samples. In each of these seven countries the DHS used almost identical multistage sampling procedures. Primary sampling units (PSUs) were selected from all administrative regions in both rural and urban areas using the probability PPS based on the most recent census of each country. Households were then selected randomly from the PSUs. Finally, based on the selection criteria, respondents were selected from the households. In the current study, seven countries with differing populations (in 2008) are included: Egypt (74.9 million), Guinea (10.3 million), Kenya (38.0 million), Mali (12.7 million), Niger (14.7 million), Senegal (12.7 million), Sierra Leone (5.5 million). In each country, sampling is based on PPS of the population. Therefore, population size has no potential influence on the current study. More details of the sampling procedures and survey methods are available in Egypt DHS 2008 ^20^, Guinea DHS 2012 ^21^, Kenya DHS 2008 ^22^, Mali DHS 2006 ^23^, Niger DHS 2012 ^24^, Senegal DHS 2010–11 ^25^, Sierra Leone DHS 2008 ^26^.

All DHSs are global initiatives to monitor demographic and health issues, including the Millennium Development Goals, in the developing countries. The respective governments, the United States Agency for International Development (USAID), and other international donor agencies finance DHSs. Macro International Incorporated (Calverton, MD) provides the technical support for conducting DHSs. DHSs are well controlled by field experts at national and international level and therefore are rigorously planned, well organized, strictly monitored, reliable, and widely used, especially in the developing countries. Experts have provided training and guidance to the field workers to develop the awareness and skills necessary to facilitate the optimal response from the respondents. Interviewers also received training in handling private responses without putting the respondents or the interviewer at risk. An interviewer’s manual was developed and provided to the field workers. The main questionnaire was initially formulated in English and then translated into local languages as needed, using appropriate scientific methods (translations and back-translations). The current study used the secondary data generated by the above-mentioned DHSs.

In the current study, female adolescents (15–19 years) were identified in the seven aforementioned sample sets and were included in the current analysis: Egypt (N=636, 4% of total women respondents), Guinea (N=1994, 21.8%), Kenya (N=1767, 20.3%), Mali (N=2791, 21.1%), Niger (N=1835, 17%), Senegal (N=3604, 23%), Sierra Leone (N=1237, 17.1%).

### Variables of interest

The main variable of interest for the current study was “Circumcision should continue or be stopped?” The respondents had the options “should continue” and “should not continue.” The current study used this as the dependent variable in all bivariate and multivariate analyses.

### Independent variables

*Place of residence:* Urban or rural area

*Religion:* Muslim and non-Muslim. The original questionnaire included other religions as well. However in the current analysis, all other religions than Muslim have been combined into new variable: non-Muslim.

*Education*: None, or primary, secondary, or higher education. In the current analysis, secondary and higher education were merged into a new variable “Secondary+.”

*Exposure to media:* The survey questionnaire had asked respondents about reading newspapers or magazines, listening to radio, and watching television. The current study created a new variable “exposure to media” by merging these three variables. Several studies have indicated that media plays an important role in the discontinuation of FGM ^12, 13, 16^.

*Economic status:* This was a composite measure of the cumulative living standard of the households, considering all economic assets (such as radios, televisions, bicycles), materials used for construction of houses, types of water access, and sanitation facilities. Principal components analysis (PCA) was used to estimate individual households on a continuous scale of relative wealth ^20– 26^. This was a standardized scale in the normal distribution with a mean of zero and a standard deviation of one. Then, using the standardized scores, wealth quintiles were created: poorest, poorer, middle, richer, and richest.

## Ethical issues

The current study uses secondary data collected from the DHS Program. The DHS had received ethical permission from the Institutional Review Board of Opinion Research Corporation (ORC) Macro International Inc.

### Statistical analyses

Prevalence was estimated for each country to reflect adolescent girls’ supportive attitudes on continuation of FGM. The proportions and chi-squared tests were used to explore the cross relationships between dependent and independent variables. Multivariate logistic regressions were performed to study the potential associations between justification of female genital mutilation and respondents’ socioeconomic factors and exposure to media. Data were analyzed using IBM SPSS version 20.0.

## Results

**Figure 1.**
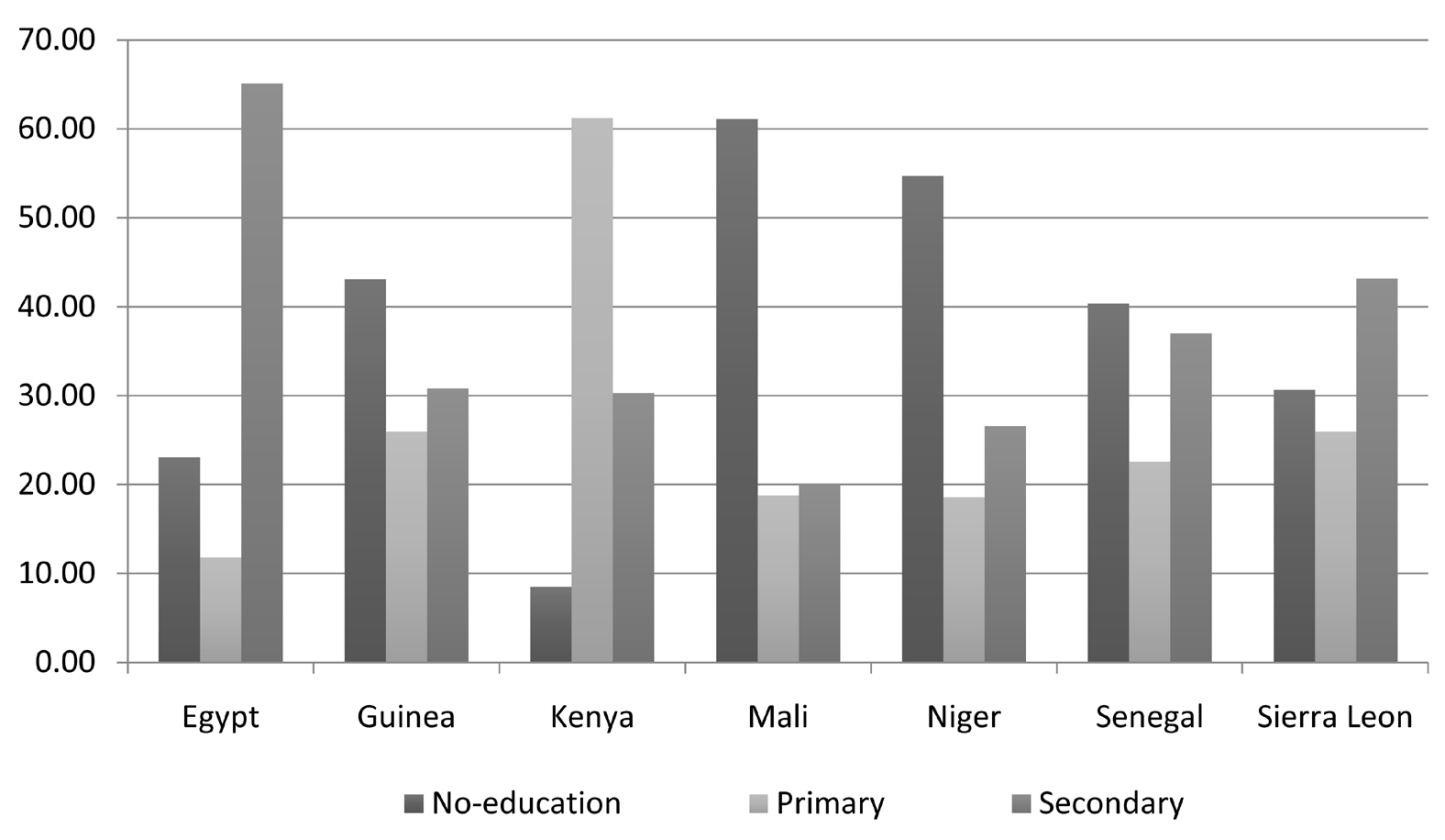
Education levels among adolescent girls (15–19 years) in seven African countries.

The sample consisted of 15–19 year-old girls in seven countries in Africa. The mean age of respondents was 17 years in six countries, and 18 years in Egypt. The proportion of respondents residing in rural areas was: in Egypt 77.4%; Guinea 55.6%; Kenya 75.5%; Mali 57.4%; Niger 62.8%; Senegal 57.5%; Sierra Leone 45.8%. Respondents were mainly of the Muslim religion in (Egypt 97.2%; Guinea 88.5%; Mali 88.9%; Senegal 94.8%). Kenya had only 17.7% Muslim respondents, and Sierra Leone had 69% Christian. Figure 1 shows the educational levels of the respondents by country.

Prevalence of FGM among all respondents of reproductive age (15–49 years) was in Egypt 94.4% (15–19 years: 91.5%); Guinea 97.9% (95.7%); Kenya 31.6% (22.7%); Mali 88.7% (88.6%); Niger 4.2% (3.5%); Senegal 40% (40.3%) Sierra Leone 90.9% (70.6%).

**Figure 2.**
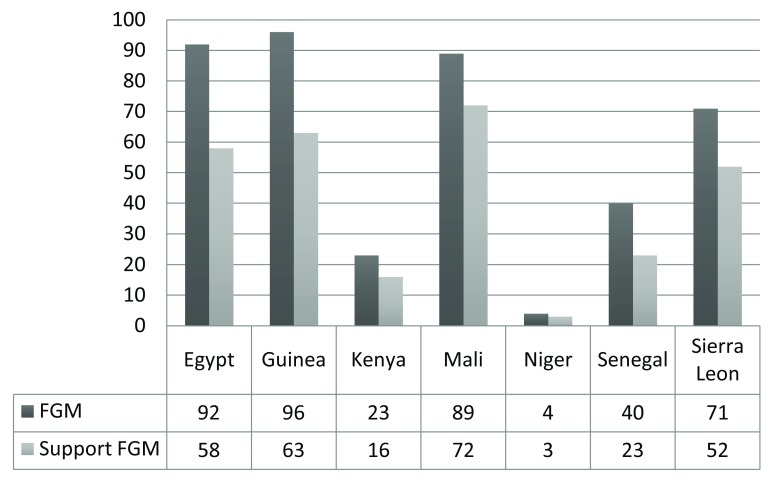
Prevalence of FGM among adolescent girls (15–19 years) and their attitudes towards continuation of FGM.

In Figure 2 we have assessed the prevalence of FGM among adolescent girls and their attitudes towards continuing FGM. Prevalence of supporting the continuation of FGM among adolescent girls was in Egypt 58%, Guinea 63%, Kenya 16%, Mali 72%, Niger 3%, Senegal 23%, and Sierra Leone 52%.

In Niger no significant differences were found between demographic factors and supporting the continuation of FGM ( Table 1) (due to the missing cases or the low proportion of support). In all other countries, girls living in rural areas or with poor economic status were more likely to support FGM. Also lower-educated girls and girls who were not exposed to media supported the continuation of FGM to a greater extent, except in Egypt.

**Table 1.** Demographic factors and economic status of adolescent (15–19 years) girls who supported continuation of FGM in Egypt (2008), Guinea (2012), Kenya (2008), Mali (2006), Niger (2012), Senegal (2010–11), Sierra Leone (2008).

	Egypt		Guinea		Kenya		Mali		Niger		Senegal		Sierra Leone	
	N	% supported	N	% supported	N	% supported	N	% supported	N	% supported	N	% supported	N	% supported
	N=636	58.0%	N=1994	69.3%	N=1767	16.2%	N=2791	71.3%	N=1835	2.8%	N=3604	22.7%	N=1237	52%
*Place of residence* Urban Rural	144 492	P=0.005 48% 61%	883 1109	P<0.001 63% 74%	411 1222	P<0.017 12% 17%	1229 1562	P=0.014 69% 73%	366 243	P=0.694 3% 2,5%	1391 1683	P<0.001 17% 27%	668 569	P<0.001 43% 62%
*Religion* Non-Muslim Muslim	18 618	P=0.095 39% 59%	230 1762	P<0.001 54% 71%	1340 291	P<0.001 8% 56%	324 2467	P<0.001 60% 73%	16 593	P=0.492 0% 3%	143 2931	P<0.001 10% 23%	384 853	P<0.001 33% 60%
*Education* No education Primary Secondary+	147 75 414	P=0.637 61% 59% 57%	859 518 615	P<0.001 77% 73% 56%	135 975 523	P<0.001 72% 12% 9%	1669 539 583	P<0.001 76% 70% 58%	282 127 200	P=0.39 3.5% 3% 1.5%	1208 668 1198	P<0.001 28% 24% 17%	381 320 536	P<0.001 68% 55% 38%
*Exposure to media* No Yes	15 621	P=0.710 53% 58%	500 1479	P<0.001 76% 67%	242 1387	P<0.001 49% 10%	349 2403	P<0.001 63% 73%	99 507	p=0.237 1% 3%	237 2837	P<0.001 37% 22%	404 813	P<0.001 64% 45%
*Economic status* Poorest Poorer Middle Richer Richest	192 182 124 87 51	P=0.003 66% 60% 55% 53% 37%	332 336 355 516 453	P<0.001 83% 74% 72% 62% 63%	351 278 325 333 346	P<0.001 34% 13% 15% 10% 8%	424 472 506 518 871	P<0.001 79% 73% 71% 66% 70%	50 56 63 101 339	P=0.302 0% 5% 5% 4% 2%	668 667 766 545 428	P<0.001 33% 29% 23% 15% 6%	174 156 200 279 428	P<0.001 66% 60% 59% 50% 41%

P-values of chi-square test.

Mali, Niger, Guinea, and Senegal had more than 40% uneducated adolescent girls (15–19 years). Almost two-thirds of respondents (15–19 years) in Kenya and Egypt had primary and secondary education respectively. Figure 1 has presented the education levels for adolescent girls (15–19 years) in seven African countries

After adjusting for all other independent variables in this study, using a logistic regression for each country independently, some results are found that are similar between the countries and one that differs between the countries ( Table 2). Religion and economic status were significantly associated with supporting the continuation of FGM in five of the participating countries. Non-Muslim participants (Odds Ratios between 0.093 and 0.502, p<0.005) were less likely to support FGM than Muslim participants in all countries except Egypt and Niger. Lower economic status (Odds Ratios between 2.226 and 7.802, p<0.005) gave a greater likelihood of supporting FGM than did the richest group in all countries but Mali. In Guinea, Kenya, Mali and Sierra Leone, having no education (Odds Ratios between 1.937 and 4.657, p<0.005) or only primary education (Odds Ratios between 1.616 and 2.108, p<0.005) resulted in a greater likelihood of supporting FGM than having secondary or higher education. In Egypt, Niger and Senegal there was no association between education and supporting FGM. The girls who stated that they had no media exposure (Odds Ratios between 1.439 and 2.837, p<0.005) showed a greater likelihood of supporting FGM in Guinea, Kenya and Senegal than those with media exposure. In Mali (Odds Ratio=0.486, p<0.005) the result showed the opposite: girls with no media exposure were less likely to support FGM than those with media exposure.

**Table 2.** Multivariate regression analysis of FGM for demographic factors and economic status in Egypt (2008), Guinea (2012), Kenya (2008), Mali (2006), Niger (2012), Senegal (2010–11), Sierra Leone (2008).

	Egypt			Guinea			Kenya			Mali			Niger			Senegal			Sierra Leone		
	ORs	95% C.I.		ORs	95% C.I.		ORs	95% C.I.		ORs	95% C.I.		ORs	95% C.I.		ORs	95% C.I.		ORs	95% C.I.	
		Lower	Upper		Lower	Upper		Lower	Upper		Lower	Upper		Lower	Upper		Lower	Upper		Lower	Upper
*Residency*																					
*Urban*	0.816	0.529	1.258	1.075	0.78	1.481	0.697	0.384	1.266	0.848	0.66	1.091	4.294	0.977	18.874	1.078	0.864	1.346	0.759	0.538	1.071
*Rural*	1.0			1.0			1.0			1.0			1.0			1.0			1.0		
*Religion*																					
*Non-Muslim*	2.426	0.913	6.449	0.38	0.279	0.522 *	0.093	0.063	0.136 *	0.502	0.389	0.646 *	0.0	0.0	-	0.319	0.18	0.565 *	0.378	0.29	0.492 *
*Muslim*	1.0			1.0			1.0			1.0			1.0			1.0			1.0		
> *Education*																					
*No education*	1.181	0.796	1.752	1.937	1.5	2.502 *	4.657	2.539	8.54 *	2.577	2.059	3.225 *	2.764	0.625	12.221	1.181	0.944	1.476	2.167	1.539	3.052 *
*Primary*	1.025	0.614	1.71	2.108	1.609	2.761 *	0.85	0.567	1.276	1.669	1.29	2.16 *	1.802	0.368	8.816	1.175	0.919	1.503	1.616	1.182	2.209 *
*Secondary ^+^*	1.0			1.0			1.0			1.0			1.0			1.0			1.0		
*Media exposure*																					
*No*	0.615	0.211	1.79	1.493	1.139	1.957 ^†^	2.837	1.812	4.442 *	0.486	0.376	0.627 *	0.223	0.027	1.824	1.439	1.071	1.935 ^†^	1.282	0.951	1.728
*Yes*	1.0			1.0			1.0			1.0			1.0			1.0			1.0		
*Economic status*																					
*Poorest*	2.989	1.438	6.209 ^†^	1.783	1.108	2.868 ^†^	1.536	0.692	3.408	1.346	0.936	1.936	0.0	0.0	-	6.747	4.264	10.676 *	1.279	0.769	2.127
*Poorer*	2.226	1.092	4.539 ^†^	1.283	0.816	2.018	1.93	0.891	4.184	0.855	0.611	1.196	7.802	1.236	49.24 ^†^	5.923	3.786	9.264 *	1.087	0.664	1.781
*Middle*	1.836	0.892	3.779	1.226	0.806	1.865	2.4	1.157	4.977 ^†^	0.759	0.549	1.048	6.736	1.071	42.374 ^†^	4.297	2.796	6.602 *	1.073	0.697	1.652
*Richer*	1.751	0.831	3.688	0.824	0.618	1.099	1.235	0.625	2.442	0.756	0.575	0.994	3.418	0.844	13.836	2.657	1.685	4.189 *	1.094	0.777	1.542
*Richest*	1.0			1.0			1.0			1.0			1.0			1.0			1.0		

1.0 denotes reference category. Significance level: “ *=> p<0.001”; “ ^†^ => p<0.050”

## Discussion

In the seven countries compared in this paper, support for FGM among adolescent girls (15–19 years of age) ranged from 3% to 71%. The countries where the support for FGM was over 50% were Egypt, Guinea, Mali, and Sierra Leone. In Senegal slightly more than 20%, and in Kenya slightly fewer than 20% of respondents supported the continuation of FGM. In Niger only 3% supported FGM; however there was a large proportion of missing cases, because the FGM prevalence is very low. The current study has indicated that adolescent girls from highly FGM-prevalent countries were more supportive of continuing FGM than those from low prevalence countries. Guinea, Kenya, Senegal, and Sierra Leone have demonstrated that exposure to media (newspapers, magazines, radio or television) has a positive effect for reducing the justification of FGM among adolescent girls. The situation in Mali is the complete opposite. Adolescent girls in Mali who are exposed to media supported the continuation of FGM more than those who are not exposed to media. The Mali study was the oldest (2006) among the seven countries, and this inconsistent finding may indicate that media did not play its expected role of protecting girls from FGM. In a recent UN report, a number of organizations have urged more media coverage to motivate the discontinuation of FGM in the affected countries ^27^. The current study echoes these calls for stronger media coverage towards discontinuation of FGM. An integrative review study has assessed 16 studies of which only five have focused on attitudes towards FGM in the country of origin ^13^. The current study uses nationally representative data from seven countries.

No education, rural residence, and lack of information ignite attitudes in favor of the continuation of FGM. Previous studies have also signaled the same findings ^12^. Empowerment is often considered an explanatory factor of supporting the discontinuation of FGM ^18, 19^. In this study the participants are 15–19 years old, live with their families, and have no socioeconomic empowerment. This could explain why the girls in the current study to some extent accepted family-oriented socioeconomic and cultural norms and supported the continuation of FGM.

Economic status and education are factors that could change attitudes toward FGM ^16, 27^. The current study similarly recommends more education and less poverty as ways to shift attitudes in favor of a discontinuation of FGM.

This study is cross-sectional in nature which leads to difficulties in assigning causal relationships between respondents’ background factors and favoring FGM. Prevalence of FGM has been determined from respondents’ self-reporting. No clinical examination was performed to verify their reported genital mutilation. Therefore, the reported prevalence could over- or underestimates the actual rate. Though all the seven DHSs have tried to select samples in all strata of the country using probability proportional to population size (PPS), after finalization of the study data, some countries have fewer respondents in the adolescent group (15–19 years). Also, countries like Niger have very low FGM prevalence. Polarization in the distribution of data (such as by religion) may lead to non-significant results. The current study used the same questionnaires for identifying the FGM prevalence and for assessing teenagers’ attitudes towards continuation of FGM using quantitative research. However, we have no answer to why the youngest generation of women supports FGM. Appropriate qualitative studies among adolescent girls exploring the reasons for such support could lead to more effective policy-making to eradicate FGM. Due to the large variations within countries, the importance ascribed to country-specific data in tailoring approaches to effectively reduce and eliminate FGM is in our view well warranted ^12, 15^.

## Policy implications

To the best of our knowledge, the current study is the first to examine the attitudes of adolescent girls (15–19 years) regarding the discontinuation of FGM in seven countries. The findings of the current study support the provision and dissemination of adequate education to eliminate FGM, and emphasize the important role of media. The study has considerable implications for policy aiming to eliminate FGM, as higher prevalence indicates a higher supportive attitude among adolescent girls. UN bodies are advocating for the elimination of FGM. However, if teenage girls support FGM, they are likely to continue the practice with their daughters when they become mothers. Previous studies have recommended educational, community, and media intervention ^1, 4, 14– 16^. The findings presented here indicate that the youngest women should be targeted for necessary interventions.

## Data availability

The DHS Program owns data used in this study. The DHS data for all the seven countries are available for researchers interested in further analyses. Researchers should contact The DHS Program and get permission to use the required data.
