# Supplementary material for: Adolescent girls’ attitudes toward female genital mutilation: a study in seven African countries
Source: F1000Res. 2018 Mar 20;7:343. [Version 1] doi: 10.12688/f1000research.14142.1 (PMC6008845; doi:10.12688/f1000research.14142.1)
Supplement: Peer review history

# Peer review: Referee response for version 1

**DOI:** 10.5256/f1000research.15382.r35488

**Published:** 2018-07-09

**Authors:** Gregory E Gilbert

**Affiliations:** 1 Learning Sciences, Adtalem Global Education, 3005 Highland Parkway, Downers Grove, IL, USA; 2 Ross University School of Medicine, Roseau, Dominica

Thank you very much for the opportunity to review this potentially important manuscript. Unfortunately, it suffers from a fatal flaw. 

 First, recommendations. This reviewer strongly recommends English language editing to improve clarity, readability, and flow of the text. The authors are to be congratulated for authoring a coherent manuscript and have done much better than this reviewer could do in (what is presumed to be a language other than their primary language). 

 The fatal flaw has to do with study design. A complex survey design was used in at least to survey the Kenyan (and I suspect other) population(s). This two-stage sampling design oversampled urban populations and undersampled those in the North East province. Not taking the sampling design into account when doing the analysis has critical implications and renders and conclusions wrong. This reviewer is unaware of SPSS survey sampling software; however, SAS has several survey procedures (PROC SURVEYMEANS, PROC SURVEYFREQ, PROC SURVEYPHREG), R (which is free) has a package for complex survey analysis (survey), and RTI (SUDAAN) all have software for handling complex surveys designs.

I have read this submission. I believe that I have an appropriate level of expertise to state that I do not consider it to be of an acceptable scientific standard, for reasons outlined above.

# Peer review: Referee response for version 1

**DOI:** 10.5256/f1000research.15382.r34761

**Published:** 2018-06-18

**Authors:** Nesrin Varol

**Affiliations:** 1 Sydney Medical School, Discipline of Obstetrics, Gynaecology and Neonatology, University of Sydney, Sydney, NSW, Australia

This is an important article that points out the most crucial aspects of the process of abandonment of FGM. Education, financial independence for women, and addressing poverty are some of the key factors to change attitudes and protect girls and women.

 Please see my other comments:

**Page 1**

**Abstract**

**Methods**

 Please delete “different” in “…seven different countries in Africa.” Countries are different by definition. 

**Results**

 Please correct grammar here and again in results section. “Prevalence of supporting the continuation……girls was 58% in Egypt, 63% in Guinea, etc…”

 Or 

 “Prevalence of supporting the continuation…..girls was: 58% Egypt, 63% Guinea, etc…..”

**Page 3**

**Introduction**

 1. “…..where FGM is common except Somalia4, as the majority of FGM procedures being carried out on young girls5.” 

 Meaning of the end of this sentence is unclear. You mean that Somalia is the only country that hasn’t ratified the Conventions. You also mean that FGM is a violation of human rights etc because it’s being performed on children / young girls. The grammar needs to be changed here. Perhaps write: 

 “It is recognized as a violation of the human rights of girls and women3, and is a violation of the Convention on the Rights of the Child, as the majority of FGM procedures being carried out on young girls5  These have been ratified by all of the countries where FGM is common except Somalia4. 

 2. “In the seven countries included in this multi-country study the estimated prevalence of FGM among girls and women between 15 and 49 years is high (over 85%) in Egypt, Guinea, Mali, and Sierra Leone.”

 You write “in the seven countries” but then only write the names of four, i.e Egypt, Guinea, Mali and Sierra Leone.

 3. “More women than men support the practice11,12 

 Please write which countries this statement refers to or detail the statement a little further. 

 4. “A multi-country comparison is a unique opportunity to analyze what demographic factors that are associated with attitudes toward the continuation of FGM.” 

 Please delete “that”. 

 5. “The aim of this study is to examine adolescent girls’ attitudes toward the continuation of female genital mutilation in association with their demographics in seven different countries in Africa. “

 Change to FGM. Once you have written an acronym, continue to use it throughout the article. Delete “different” again. 

**Page 4**

**Methods**

 “All DHSs are global initiatives to monitor demographic and health issues, including the Millennium Development Goals, in the developing countries.”

 The MDGs are the older Goals that were used. Also perhaps change from “developing” to “low-income”. So: “….including the past Millennium ….., in low-income countries.”

**Results**

 “Respondents were mainly of the Muslim religion.” 

 The religion is Islam, the people who follow Islam are Muslims. Please change to “….of the religion of Islam.”

 or 

 “…of the Islamic religion.”

**Page 5**

**Results**

 1. “Almost two-thirds of respondents (15–19 years) in Kenya and Egypt had primary and secondary education respectively. “

 Please place a comma after “…education, …”.

 2. Figures 1 & 2. “Sierra Leone” is missing the “e” at end of Leone. 

 3. “Non-Muslim participants (Odds Ratios between 0.093 and 0.502, p<0.005) were less likely to support FGM than Muslim participants in all countries except Egypt and Niger. “

 It would be interesting for the reader to know the details of the results in Egypt and Niger. It is an important result and should be nuanced in its presentation.  Are Muslims less likely to support FGM than non-Muslims? If so, please state that with the appropriate numbers.

 Page 8

 “The situation in Mali is the complete opposite. Adolescent girls in Mali who are exposed to media supported the continuation of FGM more than those who are not exposed to media. The Mali study was the oldest (2006) among the seven countries, and this inconsistent finding may indicate that media did not play its expected role of protecting girls from FGM. ”

 Yes, this is indeed an interesting finding. It would be good for you to speculate on why this may be the case. Your interpretation is part of a discussion of your findings. Perhaps the reasons may be that  the social obligation / pressure is even more entrenched in Mali? Or religious pressure?  Or even lower levels of education as compared to the other countries?

I have read this submission. I believe that I have an appropriate level of expertise to confirm that it is of an acceptable scientific standard.

# Peer review: Referee response for version 1

**DOI:** 10.5256/f1000research.15382.r33115

**Published:** 2018-05-17

**Authors:** Els Leye

**Affiliations:** 1 International Centre for Reproductive Health, Ghent University, Ghent, Belgium

The paper provides a secondary analysis of attitudes of adolescent girls towards continuation of FGM  in association with a number of variables (demographic). The paper is well written, straightforward, and shows some surprising results. 

 I think it is worthwhile publishing, as it sheds light on a particular group of interest when it comes to prevention of FGM, i.e. adolescent girls. 

 Some minor comments: .

 - use most recent prevalence data (200 million with FGM), from UNICEF 2016

 - typo in 3rd paragraph of introduction, page 3: One reason for this could be they're (not their)...

 - typo in 4th paragraph of introduction, page 3: remove 'that' in second line.

 Page 4, methods section: Millennium Development Goals have expired; you should update with the Sustainable Development Goals.

 Page 8, last sentence of first paragraph of discussion: I do not really see the relevance.

 Page 8, second column, line 5: typo: should be underestimate.

I have read this submission. I believe that I have an appropriate level of expertise to confirm that it is of an acceptable scientific standard.
